# 91例小细胞肺癌预后的回顾性多因素分析

**DOI:** 10.3779/j.issn.1009-3419.2014.08.02

**Published:** 2014-08-20

**Authors:** 影 柳, 晶 朱, 显红 刘, 影 辛, 莹 王, 颖 程

**Affiliations:** 130012 长春，吉林省肿瘤医院胸部肿瘤内科 Department of Medical Oncology, Jilin Provincial Tumor Hospital, Changchun 130012, China

**Keywords:** 肺肿瘤, TNM分期, 预后, Lung neoplasms, TNM stage, Prognosis

## Abstract

**背景与目的:**

小细胞肺癌(small cell lung cancer, SCLC)约占新发肺癌的15%-25%, 尽管新型化疗药物及放疗技术不断进展, 但预后仍较差, 为进一步探讨局限期SCLC的预后因素, 回顾性评价不同TNM分期、不同治疗模式对局限期SCLC疗效及预后的影响。

**方法:**

收集2006年1月-2012年3月期间吉林省肿瘤医院胸部肿瘤内科收治的资料完整经过序贯化放疗的局限期SCLC共91例, 根据2009年国际肺癌组织分期系统回顾性的将局限期患者分为Ⅰ期、Ⅱ期、Ⅲa期和Ⅲb期组, 比较4组的近期疗效、无进展生存期(progression-free survival, PFS)和生存期(overall survival, OS), 采用*Kaplan-Meier*法行生存分析, *Cox*比例风险模型行多因素回归分析。

**结果:**

全组患者的RR率为93.4%;全组中位PFS 14.25个月, Ⅰ期、Ⅱ期、Ⅲa期、Ⅲb期的中位PFS分别为:22.03个月、15.97个月、11.99个月和10.5个月, 其中Ⅰ期和Ⅲa期、Ⅲb期的中位PFS有统计学差异(*P* < 0.05);中位OS为19.56个月, Ⅰ期、Ⅱ期、Ⅲa期、Ⅲb期的中位生存期分别为:30.38个月、22.07个月、16.0个月和15.25个月, 其中Ⅰ期、Ⅱ期和Ⅲa期、Ⅲb期的中位生存期有统计学差异(*P* < 0.05)。Ⅲa期和Ⅲb期病例早放疗与晚放疗比较, 晚放疗组生存期优于早放疗组, 并有统计学差异(*P*=0.011)。单因素分析表明TNM分期、放疗方式和放疗前化疗周期数是影响患者预后的主要因素。*Cox*分析表明TNM分期、PS评分、放疗前化疗周期数和放疗方式是影响总生存时间的独立预后因素。

**结论:**

TNM分期是局限期SCLC的较好预后因素, 本研究显示Ⅲ期SCLC晚放疗有生存获益, 对于Ⅲa期和Ⅲb期局限期SCLC患者放疗时机的选择有待进一步研究, SCLC应用TNM分期更有利于指导治疗和预后。

## Abstract (en)

**Background and objective:**

Small cell lung cancer accounts for about 15%-25% of lung cancer, although the new chemotherapy drugs and radiation technology are continuously progressing, but the prognosis is still poor.To explore the prognostic factor of small cell lung cancer (SCLC), we study the correlation between effect, prognosis and TNM stage, various treatment mode.

**Methods:**

We collected 91 Limited-disease-SCLC patients'data From 2006 to 2012.The data were reviewed retrospectively and restaged as Ⅰ, Ⅱ, Ⅲa and Ⅲb stage groups according to the clinical staging in the seventh edition of the tumor.We compare the progression-free survival (FPS) and overall survival (OS).Survival analysis was evaluated by *Kaplan-Meier* and multivariate analysis was performed by *Cox* proportional hazards model.

**Results:**

In the whole group, patients achieved complete response and partial response, exhibited an RR of 93.4%.The median PFS was 14.25 months of which, 22.03 months in patients in stage Ⅰ, 15.97 months in stage Ⅱ, 11.99 months in stage Ⅲa and 10.5 months in stage Ⅲb (*P* < 0.05).The median overall survival (OS) was 19.56 months of which, 33.38 months in patients in stage Ⅰ, 22.07 months in stage Ⅱ, 16.0 months in stage Ⅲa and 15.52 months in stage Ⅲb (*P* < 0.05).Patients of stage Ⅲa and Ⅲb have longer survival time in earlier radiation groups then that of later radiation groups.Univariate analysis indicate stage of TNM, the pattern of radiation therapy and chemotherapy cycles before radiation therapy were significantly related to the survival in SCLC.Multivariate analysis showed that stage of TNM, ECOG (Eastern Cooperative Oncology Group) performance status, pattern of radiation therapy and cycle numbers of chemotherapy before radiation were factors correlated with survival.

**Conclusion:**

The stage of TNM may become beneficial prognostic factors in the treatment of LD-SCLC.The time of radiation therapy in stage Ⅲa and Ⅲb is of worth further investigation.

小细胞肺癌(small cell lung cancer, SCLC)约占肺癌总数的15%-20%, 我国SCLC发病率呈逐渐增高趋势, 约占新发肺癌的25%, 尽管新型化疗药物及放疗技术不断进展, 但预后仍较差。大约30%-40%的患者诊断时为局限期SCLC, 系统的化疗及放疗是局限期SCLC患者主要的治疗方式, 但大多数的SCLC患者最终出现远处转移, 预后较差^[1]^, 据统计局限期SCLC中位生存期约为14.5个月, 2年生存率约为45%^[2]^。既往根据美国退伍军人协会分期将SCLC患者分为局限期和广泛期^[3]^, 而2009年国际肺癌组织公布的新分期系统建议SCLC亦实施TNM分期系统。目前局限期患者标准治疗以同步或序贯化放疗并配合预防性脑照射(prophylactic cranial irradiation, PCI)为主要治疗方式, 不同TNM分期即使均为局限期患者其治疗模式及其他临床因素亦可能与患者疗效和预后相关。为进一步探讨局限期SCLC疗效的预测和预后相关因素, 我们回顾性的分析了2006年1月-2012年3月吉林省肿瘤医院胸部肿瘤内科收治的资料完整的经过序贯化放疗的局限期SCLC 91例, 评价不同因素对于SCLC治疗和预后的影响。

## 1 资料与方法

### 1.1 临床资料

回顾性分析2006年1月-2012年3月期间吉林省肿瘤医院胸部肿瘤内科收治的经病理学及细胞学证实的局限期SCLC患者91例, 其中男性60例(65.9%), 女性31例(34.1%); 年龄29岁-80岁, 中位年龄57岁。

2010年前的患者常规根据1973年美国退伍军人医院分期系统分为局限期, 局限期被定义为肿瘤位于一侧胸腔, 包括同侧纵隔淋巴结和双侧锁骨上淋巴结, 2010年后患者已常规按照2009年国际肺癌组织TNM分期系统进行分期, 将2010年前的病例重新进行TNM分期后进行评价。共纳入91例局限期SCLC患者, 均无恶性胸腔积液, 排除了随访少于6个月、化疗周期数少于4周期、未行胸部放疗及同步化放疗的患者, 均行序贯化放疗, 本组中Ⅰ期组:17例(均为T2aN0M0), Ⅱ期组:15例(T2aN1M0, T2bN0M0, T2aN1M0), Ⅲa期:41例(T1N2M0, T2N2M0, T3N1M0, T3N2M0), Ⅲb期:18例(T1-2N3M0, T3N3M0), 91例患者均经细胞学或病理学确定为SCLC, 分期常规根据头CT/头MRI、肺CT和骨扫描、肾上腺彩超进行检查, 没有对患者进行常规的骨髓活检。

### 1.2 治疗方法

全组病例均采用序贯化放疗治疗, 化疗的方案以标准的EP(依托泊苷联合顺铂)方案为主, 老年患者(≥70岁)以EC(依托泊苷联合卡铂)方案为主, 有2例患者因首诊化疗在外院进行而给予NP(长春瑞宾联合顺铂)方案化疗, 一线化疗平均周期数达到5.7个周期。2010年后多数局限期病例在全身化疗1个-2个周期后行序贯胸部放疗, 脑预防照射的比例亦增加, 2009年前病例多采用全身化疗3个-4个周期后行序贯胸部放疗。2010年前放疗方式以常规照射为主要治疗方式, 近4年以适形、调强放射治疗为主, 有1例患者进行了容积弧形调强放射治疗技术(volumetric-modulated arcradiotherapy, VMAT)治疗, 放射治疗应用6MV X线照射, 采用常规照射或三维适形或调强治疗, 原发灶、肺门纵隔区及双锁骨上区照射剂量范围为DT:4, 000 cGy-6, 000 cGy, 中位剂量为:50 Gy。二线治疗的比例达到了53.8%(49/91), 应用的治疗方式包括化疗(伊立替康联合顺铂、拓扑替康联合顺铂、异环磷酰胺联合EP方案等)、局部放疗、肝脏病灶射频消融治疗等。

### 1.3 疗效评价及随访

近期疗效按照RECIST 1.0版实体瘤疗效评价标准, 即完全缓解(complete response, CR)、部分缓解(partial response, PR)、稳定(stable disease, SD)、进展(progressive disease, PD), 以CR+PR计算有效率(response rate, RR), 无进展生存时间(progression-free survival, PFS)为治疗开始至疾病进展的时间, 总生存时间(overall survival, OS)为治疗开始至死亡或末次随访时间。

### 1.4 统计学分析

应用SPSS 13.0统计软件进行数据分析, 计数资料应用卡方检验, 生存分析采用*Kaplan-Meier*方法, 选择单因素分析中有统计学意义的变量进入*Cox*回归模型, 以*P* < 0.05为差异有统计学意义。

## 2 结果

### 2.1 四组间临床特征比较

对91例局限期SCLC临床资料进行分析, 全部病例均经细胞学或病理学确诊为SCLC, 根据2009年国际肺癌组织TNM分期回顾性将全组患者分为Ⅰ期组(均为T2aN0M0), Ⅱ期组(T2aN1M0, T2bN0M0, T2aN1M0), Ⅲa期组(T1N2MO, T2N2M0, T3N1M0, T3N2M0), Ⅲb期组(T1-2N3M0, T3N3M0), 在Ⅱ期中Ⅱa期8例, Ⅱb期7例(表 1), 在全组患者中Ⅲa期患者比例最高为45%(41/91)。

**1:** 91例局限期小细胞肺癌患者的临床特征 Clinical characteristics of 91 Limited-disease small cell lung cancer (SCLC) patients

Clinical data	Number (*n*=91)	Satge Ⅰ (*n*=17)	stage Ⅱ (*n*=15)	Stage Ⅲa (*n*=41)	Stage Ⅲb期 (*n*=18)	*X*^2^	*P*
Sex						0.752	0.861
Male	60	10	10	29	11		
Female	31	7	5	12	7		
Age						2.706	0.439
< 70	85	14	15	39	17		
≥70	6	3	0	2	1		
Performance score						9.025	0.172
0	18	4	4	8	2		
1	68	13	10	32	13		
2	5	0	1	1	3		
Sodium (Na) in baseline						1.315	0.726
Normal	78	16	13	34	15		
Low	13	1	2	7	3		
NSE in baseline						2.446	0.485
Normal	34	5	8	15	6		
High	57	12	7	26	12		
Median SER (mo)	4.5	3.5	4.0	4.5	4.5	3.006	0.391
Radiation mode						9.956	0.127
Conventional radiotherapy	30	7	6	15	2		
Dimensional conformal or intensity modulated radiation therapy	61	10	9	26	16		
Chemotherapy prior radiation therapy						5.486	0.139
1-2 cycles	35	9	3	18	5		
3-4 cycles	56	8	12	23	13		
Prophylactic cranial irradiation	22	4	5	11	2	1.926	0.588
Second line treatment	49	9	9	24	7	2.273	0.518
NSE:neuron-specific enolase; SER:start of any treatment to end of radiation therapy.							

### 2.2 TNM分期、治疗模式和近期疗效及远期疗效

91例局限期SCLC患者共完成化疗526个周期, 每例患者至少进行4周期化疗, 全组一线化放疗CR:32例, PR:53例, SD:4例, PD:2例, RR:93.4%。中位随访30个月, 全组中位PFS为14.25个月, 中位OS为19.56个月。根据不同TNM分期进行分层, 显示Ⅰ期、Ⅱ期、Ⅲa期、Ⅲb期的中位PFS分别为:22.03个月, 15.97个月, 11.99个月和10.50个月, 其中Ⅰ期和Ⅲa期、Ⅲb期的中位PFS有统计学差异(*P* < 0.05), Ⅱ期、Ⅲa期和Ⅲb期的PFS无统计学差异。中位生存期分别为:30.38个月, 22.07个月, 16.00个月和15.25个月, 其中Ⅰ期、Ⅱ期和Ⅲa期、Ⅲb期的中位生存期有统计学差异(*P* < 0.05), Ⅲa期、Ⅲb期的中位生存期无统计学差异(*P*=0.77)(表 2, 图 1)。

**2:** 不同TNM分期局限期SCLC近期及远期疗效比较 Comparison of short-term and long-term effect in different TNM stage in limited-disease SCLC

TNM	N	CR	PR	SD	PD	RR (%)	Median PFS (mo)	Median OS (mo) (95%CI)
Stage Ⅰ	17	8	9	0	0	100	22.0	30.3
Stage Ⅱ	15	4	10	1	0	93.3	15.9	22.1
Stage Ⅲa	41	14	22	3	2	87.8	11.9	16.0
Stage Ⅲb期	18	6	12	0	0	100	10.5	15.3
TNM:the TNM (Tumor, Node, Metastasis) stage of lung cancer; CR:complete response; PR:partial response; SD:stable disease; PD:progressive disease; RR:response rate; PFS:progression-free survival.								

**1:**
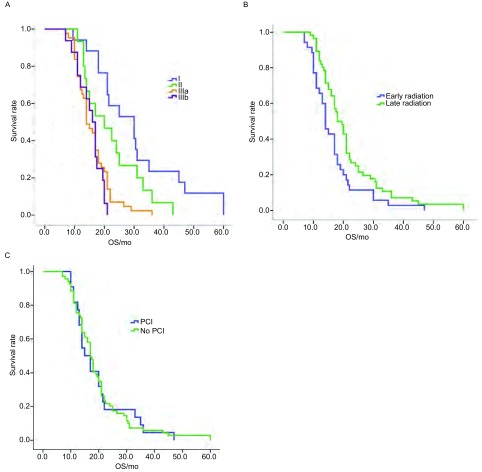
生存曲线。A:91例局限期SCLC不同TNM分期*Kaplan-Meier*生存曲线; B:Ⅲa期和Ⅲb期病例不同治疗模式(早放疗与晚放疗)生存比较; C:预防脑照射和未行预防性脑照射生存期比较。 Survival curve.A:*Kaplan-Meier* survival curve in different TNM stage; B:Survival comparison with stage Ⅲa and stage Ⅲb treated by different radiotherapy mode; C:Survival comparison with PCI (prophylactic cranial irradiation) and no PCI.

我们界定放疗前化疗1个-2个周期为早放疗, 3个-6个周期为晚放疗, 结果显示晚放疗组生存期优于早放疗组, 早放疗组的中位OS为16.48个月, 晚放疗组为:21.47个月, 具有统计学差异(*P*=0.014), 进一步分层分析显示根据Ⅰ期、Ⅱ期病例早放疗与晚放疗生存期无统计学差异(*P*=0.072), Ⅲa期和Ⅲb期病例早放疗与晚放疗生存期有统计学差异(*P*=0.011)(图 1B)。全组患者22例行预防性脑照射, 与未行脑预防性照射患者生存期无显著统计学差异(*P*=0.941)(图 1C)。

### 2.3 复发和转移方式

表 3列出了复发和转移的部位, 21个患者为局部复发, 47个患者为远处转移, 最常见的复发部位为脑, 其次为骨。

**3:** 91例局限期SCLC复发及转移部位 Relapsing and metastasis lesions

Lesions	*n* (%)
Relapse	21 (30.9)
Relapse within the radiation field	19 (28.0)
Relapse outside the radiation field	2 (2.9)
Metastasis	47 (69.11)
Brain (meninx and cerebellum)	15 (22.6)
Bone	5 (7.4)
Liver	10 (14.7)
Adrenal gland	4 (5.9)
Lymphoma	9 (13.2)
Subcutaneous	2 (2.9)
Others (thyroid gland, lung)	2 (2.9)

### 2.4 91例局限期SCLC影响生存的单因素分析

对全组91例SCLC患者预后相关因素的生存期进行比较, 单因素分析表明TNM分期、放疗方式和放疗前化疗周期数是影响患者预后的主要因素。而性别、年龄、PS评分、基线血NA、NSE值、预防性脑照射等与预后相关性无统计学意义(表 4)。

**4:** 91例局限期SCLC的总生存期的单因素分析 Single factor analysis of overall survival in 91 Limited-disease SCLC patients

Factors	Number (*n*=91)	Median survival time (mo)	*P*
Sex			0.629
Male	60	19.9	
Female	31	18.8	
Age (yr)			0.528
< 70	85	19.3	
≥70	6	21.1	
PS			0.192
0	18	21.1	
1	68	19.7	
2	5	11.8	
Stage			< 0.001
Ⅰ	17	30.4	
Ⅱ	15	22.7	
Ⅲa	41	16.0	
Ⅲb期	18	15.3	
Sodium (Na) in baseline			0.236
Normal	78	20.0	
Low	13	16.1	
NSE in baseline			0.672
Normal	34	20.2	
High	57	19.2	
Radiation mode			0.011
Conventional radiotherapy	30	22.7	
Dimensional conformal radiation therapy	51	19.3	
Intensity modulated radiation therapy	10	11.7	
Chemotherapy prior radiation therapy			0.024
1-2 cycles	35	16.5	
3-4 cycles	56	21.5	
Preventive whole brain radiotherapy	22		0.940
Yes		19.4	
No		19.6	

### 2.5 影响局限期SCLC OS的多因素分析

*Cox*分析表明分期、PS评分、放疗前化疗周期数和放疗方式是影响总生存时间的独立预后因素(表 5)。

**5:** *Cox*回归分析 *Cox* regression analysis

	*P*	HR	95%CI for Exp(B)	
			Uper	Lower
Age	0.938	1.027	0.523	2.017
PS	0.016			
PS (1)	0.005	0.185	0.056	0.609
PS (2)	0.058	0.342	0.113	1.035
Stage	0.002			
Stage (1)	0.005	0.291	0.124	0.683
Stage (2)	0.205	0.577	0.246	1.351
Stage (3)	0.894	1.046	0.542	2.017
Chemotherapy prior radiation therapy	0.024	1.763	1.076	2.888
Radiation mode	0.008			
Radiation mode (1)	0.002	0.202	0.074	0.552
Radiation mode (2)	0.007	0.318	0.138	0.733
PCI	0.911	0.965	0.517	1.802
PS(1):PS 0 *vs* PS 1;PS(2):PS 0 *vs* PS 2;Stage(1):Stage Ⅰ *vs* Stage Ⅱ; Stage(2):Stage Ⅰ *vs* Stage Ⅲa; Stage(3):Stage Ⅰ *vs* Stage Ⅲb; Radiation mode(1):Conventional radiotherapy *vs* Dimensional conformal radiation therapy; Radiation mode(2):Conventional radiotherapy *vs* Intensity modulated radiation therapy.				

## 3 讨论

中国人群SCLC发病率呈逐年升高的趋势, 虽然各种新型治疗药物及治疗技术不断进展, 但据统计近二十年SCLC的生存并未得到明显改善^[4]^, 其治疗仍是困扰临床医生的热点和难点。SCLC和其他恶性肿瘤一样秉承分期治疗的原则, 按照目前美国NCCN指南及卫生部肺癌诊疗规范, 局限期SCLC的治疗以同步或序贯化放疗, 对于治疗达到缓解的患者辅以预防性脑照射为主要治疗策略。但在临床实践中发现, 局限期SCLC患者给予系统的化放疗其预后亦不相同, 是否不同的临床因素影响局限期SCLC的预后值得进一步探讨, 尤其考虑到化放疗的毒性, 是否可将局限期SCLC患者根据临床因素进行分层, 根据治愈的可能性大小采用不同的个体化治疗策略, 可能将有助于进一步改善局限期SCLC预后并提高患者生活质量。

在各项可能影响局限期SCLC的临床因素中, 肿瘤的大小和局部淋巴结转移情况可能是最重要的影响预后因素^[5]^。2007年国际肺癌组织肺癌分期项目应用第七版肺癌TNM分期对SCLC的预后进行了回顾性评价^[6]^。共纳入了12, 620个病例, 其中适于行TNM分期有8, 088例患者, 结果显示生存和T和N均明显相关, 尤其是对于没有纵隔和锁骨上淋巴结的患者生存差异更明显, 由此国际肺癌组织建议SCLC患者应用第7版的TNM分期系统进行分期^[7]^。我们回顾性的将既往按照美国退伍军人协会分期法分期的局限期SCLC患者重新按照第7版TNM分期法进行重新分期, 并按照Ⅰ期、Ⅱ期, Ⅲa期和Ⅲb期进行分层, 因所纳入的患者均为Ⅰb期, 且Ⅱa和Ⅱb期例数均较少, 故未再进一步分层, 而在全部病例中Ⅲa期的比例最高, 约占患者总数的45%(41/91), 结果显示四组的近期有效率类似, 总体RR率为93.4%, 各组之间无统计学差异。全组中位PFS为14.25个月, Ⅰ期、Ⅱ期、Ⅲa期、Ⅲb期的中位PFS分别为:22.03个月、15.97个月、11.99个月和10.50个月, 其中Ⅰ期和Ⅲa期、Ⅲb期的中位PFS有统计学差异(*P* < 0.05), Ⅱ期、Ⅲa期和Ⅲb期的PFS无统计学差异; 中位OS为19.56个月, Ⅰ期、Ⅱ期、Ⅲa期、Ⅲb期的中位生存期分别为:30.38个月, 22.07个月, 16.0个月和15.25个月, 其中Ⅰ期、Ⅱ期和Ⅲa期、Ⅲb期的中位生存期有统计学差异(*P* < 0.05), Ⅲa期、Ⅲb期的中位生存期无统计学差异(*P*=0.77)。以上结果提示TNM分期与患者的PFS和总OS均相关, 尤其是OS, 说明对于局限期SCLC的患者进行更进一步的TNM分期的必要性, 尤其Ⅰ期和Ⅲ期患者比较无论PFS和OS均有明显统计学差异, 提示我们对于不同TNM分期的局限期SCLC患者可能应采取不同的治疗模式和策略, 以改善其预后。

既往放疗在SCLC中的作用受到争议, 1992年2项*meta*分析证实了胸部放疗可以减少局部复发和延长生存, 这项研究结果显示同步化放疗和加速超分割放疗安全性好, 5年生存率可提高到26%^[8, 9]^, 对于完全缓解的患者推荐行PCI, 一项荟萃分析显示完全缓解的患者行PCI后3年生存率可达到5.4%^[10]^, 另外有研究认为放疗的剂量与肿瘤直径的比值与局限期SCLC的预后相关^[11]^, 本组纳入的病例均为行胸部放疗的患者, 采用常规照射或三维适形或调强治疗, 原发灶、肺门纵隔区及双锁骨上区照射剂量范围为DT:4, 000 cGy-6, 000 cGy, 中位剂量为:50 Gy。虽然临床证据提示局限期SCLC的生存由于放疗的参与得到提高, 但是放疗时机一直受到质疑, *meta*分析揭示了当应用含铂方案化疗SER是最重要的预测因素^[12, 13]^, 一项Ⅲ期临床研究纳入231例局限期SCLC患者, 显示了相似的趋势支持短的从治疗开始到放疗结束的时间间隔(start of any treatment to end of radiation therapy, SER), 但在总OS上没有差别。目前指南及卫生部肺癌诊疗规范建议在化疗1个-2个周期进行放疗, 但在2012年的ASCO年会上, 一项来自韩国的EP化疗第1周期或第3周期联合TRT以明确局限期SCLC最佳放疗时机的Ⅲ期研究公布, 该研究结果提示延迟组(*n*=108):中位OS为26.8个月, 起始组(*n*=111):中位OS为24.1个月; 延迟组中位PFS(*n*=108):11.2个月, 起始组中位PFS(*n*=111):中位12.4个月, 提示延长组在OS和CR与起始组相似, 但延迟组发热性中性粒细胞减少的发生率更低^[14]^。我们的研究获得了相似的结果, 全组晚放疗与早放疗比较OS优势更明显, 分层分析显示Ⅰ期、Ⅱ期病例早放疗与晚放疗生存期无统计学差异(*P*=0.072), Ⅲa期和Ⅲb期病例早放疗与晚放疗生存期有统计学差异(*P*=0.011), 提示对于局限期中分期较晚的病例是否可给予化疗3个-4个周期后放疗, 可能会进一步减少远处转移而提高生存期, 值得进一步探讨。在本组研究中预防脑照射的实施与预后无差异, 考虑为纳入的病例数及在临床中能够同意实施PCI的患者数还相对较少, 在本组中尽管初治RR率达到了93.4%, 但仅有22/91实施了PCI。另外, 肿瘤标志物也被认为和患者的预后相关, 大多数这些研究和NSE和Pro-GRP有关, NSE经常在广泛期疾病增高, NSE指标增高往往预示疾病扩散^[15]^。所以, NSE可能是疾病扩散的预示指标, 我们的研究也显示基线NSE高的患者远处转移的发生率为73.08%(17/26), 而基线NSE正常的患者远处转移的发生率为50%(7/14), 患者拥有高水平的NSE有可能提示具有远处转移作为第一复发部位的倾向。

本研究结果提示即使同为局限期SCLC患者经序贯化放疗其预后亦不相同, 评价这些预测及预后因素可为制定患者的治疗策略提供依据, 其中TNM分期是较好的预后因素, 临床中针对Ⅰ期、Ⅱ期患者其可能具有更大可治愈的潜能, 应考虑加强治疗强度, 包括放射的方式和剂量, 也包括手术治疗的可能, 以进一步提高生存。而对于Ⅲ期患者、老年及PS评分较差患者应用治疗密集方案对生存期延长有限, 反而有可能增加治疗的风险和死亡率, 尤其Ⅲ期的患者已具有远处转移的潜在可能, 放疗时机的选择是否可能有别于Ⅰ期、Ⅱ期患者值得进一步研究, 当然选择的偏倚也影响了本项研究的结果, 应进一步开展前瞻性大样本研究以不断推进局限期SCLC治疗策略的发展。
